# Supplementary material for: A Bayesian approach to construct confidence intervals for comparing the rainfall dispersion in Thailand
Source: PeerJ. 2020 Feb 11;8:e8502. doi: 10.7717/peerj.8502 (PMC7020819; doi:10.7717/peerj.8502)
Supplement: Supplemental Information 2 [file peerj-08-8502-s002.docx]

**Dataset S1**. Weekly rainfall records in northern and northeastern regions during 2-8 July 2018

| Northern | |  | Northeastern | | | | | | |
| --- | --- | --- | --- | --- | --- | --- | --- | --- | --- |
| 5.0 | 0 |  | 50.8 | 0 | 128.8 | 60.5 | 0 | 11.7 | 14.2 |
| 11.7 | 0 |  | 20 | 7.0 | 149.2 | 68.1 | 1.4 | 38.3 | 0 |
| 0 | 10.0 |  | 19.4 | 11.0 | 78.7 | 65.2 | 6.4 | 0 | 1 |
| 0 | 0 |  | 14.0 | 10.0 | 68.0 | 29 | 2.3 | 53.4 | 3.4 |
| 0 | 4.8 |  | 0 | 8.0 | 67.7 | 33.1 | 0 | 141.9 | 25.5 |
| 88.8 | 21.0 |  | 0 | 25.0 | 48.3 | 65.4 | 0 | 23.8 | 0 |
| 43 | 0 |  | 15.2 | 2.4 | 156.5 | 83.0 | 1.5 | 41.6 | 4.5 |
| 17.2 | 30.0 |  | 40.0 | 30.0 | 64.5 | 135.0 | 17.8 | 76.1 | 11.2 |
| 7.6 | 18.2 |  | 12.5 | 0 | 98.0 | 0 | 0 | 10.5 | 6.5 |
| 0 | 2.8 |  | 0 | 7.0 | 5.3 | 7.4 | 17.8 | 0 | 0 |
| 0 | 25.9 |  | 4.5 | 21.2 | 10.8 | 0 | 0 | 0 | 0 |
| 6.2 | 77.0 |  | 0 | 0 | 0 | 8.2 | 62.9 | 57.5 | 0 |
| 0 | 0 |  | 3.3 | 4.0 | 0 | 0 | 17.4 | 0.5 | 0 |
| 0 | 27.2 |  | 6.7 | 30.7 | 5.2 | 0 | 0 | 2.8 | 0 |
| 9.7 | 0 |  | 0.4 | 21.5 | 12.3 | 2.0 | 0 | 35.1 | 0 |
| 7.4 | 30.1 |  | 6.4 | 26.8 | 1.3 | 0 | 0 | 0 | 0 |
| 7.4 | 17.6 |  | 3.4 | 5.1 | 11.0 | 0 | 0 | 12.5 | 0 |
| 0 | 0 |  | 7.8 | 9.7 | 7.0 | 8.0 | 0 | 22 | 0 |
| 0 | 12.1 |  | 2.5 | 27.0 | 13.0 | 0 | 0 | 8.4 | 0 |
| 1.2 | 42.4 |  | 0.7 | 33.2 | 0 | 0 | 0 | 4.5 | 6.4 |
| 0 | 0 |  | 10.6 | 43.2 | 20.0 | 0 | 38.0 | 37.0 | 0 |
| 0 | 14.5 |  | 0 | 12.8 | 19.2 | 14.5 | 4.2 | 17.5 | 0 |
| 14.3 | 25.3 |  | 4.4 | 0 | 3.6 | 30.3 | 10.0 | 10.3 | 0 |
| 7.1 | 22.1 |  | 5.4 | 8.0 | 0 | 31.5 | 23.1 | 18.8 | 0 |
| 5.3 | 3.4 |  | 5.4 | 4.6 | 1.1 | 11.5 | 76.1 | 8.0 |  |
| 16.2 | 0 |  | 0 | 3.5 | 0 | 20 | 12.0 | 0 |  |
| 14.5 | 9.4 |  | 4.7 | 1.4 | 9.3 | 14.5 | 25.5 | 0 |  |
| 2.4 | 20.1 |  | 30 | 0.5 | 0 | 4.0 | 45.0 | 0 |  |
| 30 | 20.2 |  | 27 | 15.2 | 13.5 | 10 | 28.7 | 7.2 |  |
| 0 | 19.8 |  | 0 | 99.5 | 0 | 8.0 | 0 | 0 |  |
| 2.8 | 0 |  | 31.0 | 5.9 | 4.2 | 0 | 49.5 | 60.0 |  |
| Source: Thai Meteorological Department | | | | |  |  |  |  |  |
| <https://www.tmd.go.th/services/weekly_report.php> | | | | | |  |  |  |  |
